# Emission Characteristics of Organic Light-Emitting Diodes and Organic Thin-Films with Planar and Corrugated Structures

**DOI:** 10.3390/ijms11041527

**Published:** 2010-04-12

**Authors:** Mao-Kuo Wei, Chii-Wann Lin, Chih-Chung Yang, Yean-Woei Kiang, Jiun-Haw Lee, Hoang-Yan Lin

**Affiliations:** 1 Department of Materials Science and Engineering, National Dong Hwa University, No. 1, Sec. 2, Da Hsueh Rd., Shoufeng, Hualien 97401,Taiwan; 2 Institute of Opto-Electronic Engineering, National Dong Hwa University, No. 1, Sec. 2, Da Hsueh Rd., Shoufeng, Hualien 97401, Taiwan; 3 Department of Electrical Engineering, National Taiwan University, No. 1, Sec. 4, Roosevelt Rd., Taipei 10617, Taiwan; 4 Institute of Biomedical Engineering, National Taiwan University, No. 1, Sec. 4, Roosevelt Rd., Taipei 10617, Taiwan; 5 Graduate Institute of Photonics and Optoelectronics, National Taiwan University, No. 1, Sec. 4, Roosevelt Rd., Taipei 10617, Taiwan; 6 Graduate Institute of Communication Engineering, National Taiwan University, No. 1, Sec. 4, Roosevelt Rd., Taipei 10617, Taiwan

**Keywords:** OLED, extraction efficiency, microstructure

## Abstract

In this paper, we review the emission characteristics from organic light-emitting diodes (OLEDs) and organic molecular thin films with planar and corrugated structures. In a planar thin film structure, light emission from OLEDs was strongly influenced by the interference effect. With suitable design of microcavity structure and layer thicknesses adjustment, optical characteristics can be engineered to achieve high optical intensity, suitable emission wavelength, and broad viewing angles. To increase the extraction efficiency from OLEDs and organic thin-films, corrugated structure with micro- and nano-scale were applied. Microstructures can effectively redirects the waveguiding light in the substrate outside the device. For nanostructures, it is also possible to couple out the organic and plasmonic modes, not only the substrate mode.

## 1. Introduction

Organic materials have attracted much attention for applications in electronic and optoelectronic devices, such as organic transistors, organic solar cells and organic light-emitting devices [1–4]. The organic light-emitting diode (OLED) is one of the most promising technologies for display and lighting applications [5,6]. Compared with existing liquid crystal display (LCD) technology, OLED exhibits the advantages of self-emission, wide viewing angle, fast response time, simple structure, and low driving voltage. The semiconductor light-emitting diode (LED) is also an emerging lighting technology [7,8]. However, epitaxial growth on specific substrates is needed for satisfying lattice match conditions. Such a crystallized semiconductor structure limits the substrate size, and hence LED is typically regarded as a point light source. On the other hand, for OLED technology, the fabrication conditions are similar to those of LCDs. That is, the device is fabricated on a huge glass substrate as a flat panel display, and can be regarded as a planar light-source [9]. OLED is a self-emissive display, and no extra backlight unit is needed, which makes the process flow easier, compared to LCD technology. Besides, OLED fabrication is typically a low temperature process, which is suitable for flexible optoelectronics applications [10].

**Figure 1.**
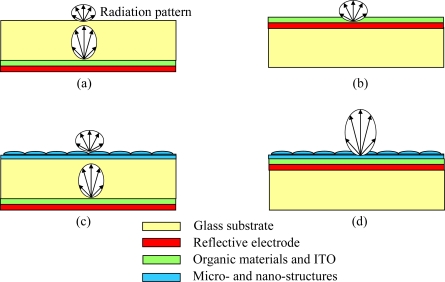
Device structures of (**a**) planar bottom-emitting, (**b**) planar top-emitting, (**c**) non-planar bottom-emitting, and (**d**) non-planar top-emitting OLEDs.

Conventionally, an OLED is fabricated on a glass substrate. Organic thin-films are sandwiched by a transparent indium tin oxide (ITO) anode and a reflective metal cathode, which is also called bottom-emission OLED, as shown in Figure 1(a) [11]. By applying a voltage (typically <10 V) to such a device, carriers are injected into the device and recombine to give light. Photons generated from the organic layers propagate through the glass substrate and radiate out to the air for light emission. Emission wavelength of OLED is determined by the organic material, as well as the device configuration. Because the conductivity of organic material is quite low, layer thickness of total organic thin films was limited to 100 to 200 nm for driving an OLED with reasonably low voltage (ex: <10 V) [12]. Considering the refractive index (n) of ITO and organic materials (typically 1.6 to 2.2 at visible region), an OLED can be regarded as a microcavity with the effective optical length comparable to the visible wavelength (from 380 to 780 nm), which results in a strong interference effect within such a device (including ITO anode, organic thin films, and reflective cathode). With fixed organic materials, the optical intensity of OLEDs can be varied with different layer thicknesses, depending on constructive or destructive interferences [13,14]. Such an interference effect is sensitive to the viewing angle and wavelength. For display application, emission from normal direction may be most important. On the other hand, one may need to optimize the total flux of light emission. Besides, the optimized cavity structure for a certain wavelength (ex: 600 nm for red light emission) may be not the best choice for another wavelength (ex: 470 nm for blue one), which results in difficulties in designing a broadband emission, such as white OLED. Optical loss in ITO and organic layers was typically called organic mode. Light may also couple to surface plasmon (SP) mode of metal cathode which relaxes the energy nonradiatively. Besides, as other EL devices, light was waveguided in the substrate, and it was called substrate mode [15]. Thickness of glass substrate is typically several hundreds of μm for providing enough mechanical strength. Refractive index of glass substrate is about 1.5, which is smaller than organic and ITO, but larger than the air (n = 1). An alternative device configuration to completely remove the substrate mode is to use a reflective anode, and a transparent (or semi-transparent) cathode, so that the light emits from the cathode side to the air, which is called top-emission OLED, as shown in Figure 1(b). Waveguiding in substrate and organic modes can be possibly coupled out *via* the introduction of a non-planar structure. Similar to the concept used in semiconductor LEDs, a hemispherical lens on a small OLED is a good example of how to efficiently couple out the light rays in the glass substrate mode. There are two obvious drawbacks for this configuration. The first is its bulky structure. Besides, it is only applicable for light source applications, rather than a real display. By using some micro- or nano-structures (such as microlense arrays, micro-pyramids, diffusers…), it is possible to shrink the thickness as a film and attach on the OLED for lighting and display applications [16–18]. Dimension of microstructures are typically much larger than the wavelength from OLED emission (*i.e.*, visible light: 380 to 780 nm), and can be treated with light ray optics. On the other hand, when a nano-structure is applied to the OLED, a rigorous treatment based on electromagnetic wave propagation may be needed. For a bottom-emission OLED, extraction efficiency can be greatly improved with attachment of optical films with microstructures, due to the extraction of glass substrate mode, as shown in Figure 1(c). Although possible, it is relatively difficult to extract the organic and SP modes, as shown in Figure 1(d). Because the thicknesses of organic thin films are in the order of hundreds nm, the corrugated structure introduced near the device may seriously affect the electrical characteristics of OLED. In summary, to improve the extraction efficiency, one may try to reduce the organic, SP, and substrate mode, which can be achieved by suitable thickness adjustment, thin-film design, and non-planar structure. In the following review, we will first introduce the operation principles of OLED in Section 2. Then, optimization of optical characteristics of planar OLEDs with top- and bottom-emission configurations will be discussed in Section 3. In Section 4, we will illustrate some non-planar structures to couple out the photons of the OLEDs. Finally, a summary will be given.

## 2. Operation Principle of OLEDs

**Figure 2.**
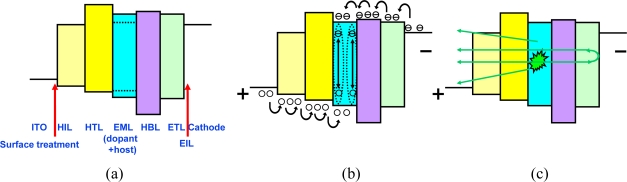
(**a**) Energy diagram of multi-layer OLED, (**b**) electronic process and exciton formation under forward bias, and (**c**) photon generation and propagation.

Although electroluminescence (EL) from organic materials was observed in 1960 by Pope *et al.*, the driving voltage then was as high as hundreds of Volts, due to the thick organic layers [19]. In 1987, Tang *et al.* proposed a OLED with two-layer organic layers, consisting of an aromatic diamine and metal chelate as the hole-transporting layer (HTL) and electron-transporting layer (ETL) materials, respectively, sandwiched by ITO anode and Mg:Ag alloy cathode [20]. Thicknesses of HTL and ETL were 75 and 60 nm, respectively, which reduced the driving voltage to less than 10 V. Under electrical current driving, hole and electrons injected from the anode and cathode, and then transported through HTL and ETL, respectively. Because of differences in energy level and carrier mobility of HTL and ETL, a hole is injected into the HTL and recombines with the electron inside the ETL near the HTL/ETL interface, forming exitons [21]. The recombination zone can be also adjusted by tuning the material mobility for achieving electron-hole balance [22–24]. Excitons are neutral and hence will not drift under electrical field. Instead, they diffuse from high to low concentration, *i.e.*, away the HTL/ETL interface. Then excitons recombine to give light. Part of the photons propagate toward the HTL, ITO, glass substrate and out of the device and part of them propagate toward the metal cathode and reflect back [25]. In this EL process, the workfunction of the anode (and cathode) should be high (and low, respectively) to facilitate hole (and electron) injection. Alkali and alkaline metals are suitable choices for cathode materials. However, due to the high reactivity of such materials, alloys (such as Mg:Ag) or double layer structures (Li:Al) were typically used. An electron injection layer (EIL), such as LiF or Cs_2_CO_2_, was introduced for better injection efficiency between a stable cathode (Al) and ETL [26]. For hole injection, the workfunction should be as high as possible. Typically, the workfunction of an ITO is about 4.6 eV, which can be further improved by CFx or oxygen plasma treatment [27,28]. An additional hole injection layer was also added to reduce the energy barrier, which also helped to increase device stability [29]. Organic dyes were widely used in laser applications due to their high internal quantum efficiency. By doping some high efficiency emitter into the organic matrix exhibiting good carrier transport characteristics we can simultaneously fulfill the requirement for better conductivity and high optical efficiency [30–32]. In organic materials, hole mobility is typically much higher than electron mobility. Such a difference in carrier mobility results in tunneling of holes to the cathode without recombination, which in turns reduces the current efficiency and shortens the lifetime [33]. Sometimes, a hole blocking layer (HBL) was inserted between the EML and ETL to block the holes from leaking to the cathode [34]. Figure 2(a) shows the workfunction of electrodes and energy levels of organic layers. Figure 2(b) shows the electrical process and exciton formation, and Figure 2(c) illustrates the photon generation and propagation inside the OLED. Conductivity of organic materials is typically low, not only because of low mobility, but also the low carrier concentration. By adding a Lewis acid into the HTL, electrons would be attracted which releases free holes for conduction and shifts the Fermi level toward highest occupied molecular orbital (HOMO) level [35]. Such a concept is similar to the p-type doping in semiconductor material. On the other hand, by doping alkali metals directly into ETL increases the electron concentrations, which is called the metal dopant technology [36]. Not only improving the transport characteristics, anode/HTL and ETL/cathode are modified from Schottky to Ohmic contact which facilitates the carrier injection.

## 3. Device Design of Planar OLEDs

**Figure 3.**
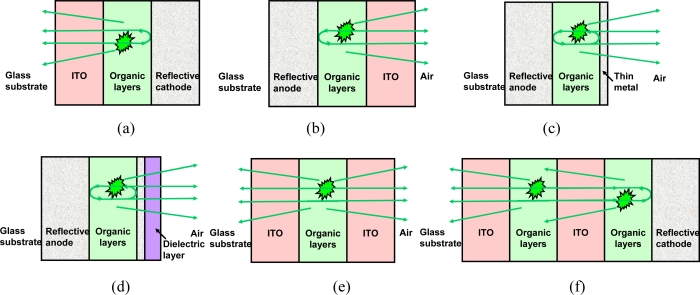
OLED with different device configuration: (**a**) bottom emission, (**b**) top emission with ITO cathode, (**c**) top emission with thin metal cathode, (**d**) top emission with thin metal cathode and dielectric layer, (**e**) transparent OLED, and (**f**) tandem OLED (2-stack).

As mentioned above, photons are generated inside the organic layers with n = 1.6–2.0 in the visible range, sandwiched by two electrodes. As shown in Figure 3(a), for a bottom-emission OLED, the ITO anode (n = 1.8–2.0) is transparent and the Al cathode is reflective. The other side of the ITO is the glass substrate (n = 1.5). Because the refractive index difference, there is also some reflection at ITO/organic, ITO/glass substrate, and substrate/air interface. Refractive index (n) and absorption coefficient (k) of organic thin films play important roles in optimizing OLEDs’ performances. By using ellipsometry and oscillator models, those important optical parameters can be extracted [37]. In a planar structure, it can be viewed as a Fabry-Perot cavity [38]. In a bottom-emission OLED, the glass substrate is very thick (typically hundreds of μm) and the free spectral range is too small to be resolved. With a simplified model, the glass substrate is first treated as infinitely thick for obtaining the emission characteristics in the glass, and then the propagation paths from the glass to the air are calculated by using ray-optics. However it may result in some errors, especially at larger viewing angles and a more rigorous models is required [39,40] When varying the ITO and organic layer thicknesses, the optical intensity and emission wavelength change a lot. Because the reflection is most significant at the reflective cathode, the distance between recombination zone to the cathode plays a dominant role in determining the optical intensity, while varying layer thickness at the anode side is still needed for optimizing the whole intensity, together with tuning of the emission color [41]. Because an OLED is sensitive to ambient environment (such as water and oxygen), encapsulation under inert gas conditions (such as N_2_ or Ar) is needed right after the thin-film process [42]. Besides, it is not possible to perform a high temperature process after the thin-film formation, which results in the crystallization of organic materials [43]. Hence, for a display application, thin-film transistors (TFT) would be fabricated underneath the OLED [44]. For a bottom-emission OLED, the aperture ratio would be limited because the TFTs occupy a certain area. Top-emission OLED uses a reflective anode to optically isolate the TFTs and OLEDs, which improves the aperture ratio for higher resolution and luminance [45]. Besides, a top-emission OLED also exhibits the potential advantages of higher extraction efficiency because there is no substrate mode. As shown in Figure 3(b), in a top-emission OLED, one side of the cathode is organic layers and the other one is air (n = 1). Hence, the refractive index change on the cathode side of a top-emission OLED is larger than that of a bottom-emission one where the anode contacts with a glass substrate (n = 1.5). That means the cavity effect is stronger in a top-emission OLED than in a bottom-emission one. With a stronger cavity effect, the optical intensity depends more on the viewing angle and the emission wavelength. Typically, emission from a top-emission OLED is more directional with a narrower spectra compared to those of bottom-emission one. Besides, such a strong cavity effect in a top-emission OLED modifies the spontaneous emission rate, called the Purcell effect, which in turns to affect the optical characteristics [46,47]. By doping dopants with different emission wavelengths at different positions and controlling the recombination zone with different voltage, it is also possible to tune the emission color in a single OLED under different bias conditions [48]. Similarly, one may engineer the optical characteristics of the anode and cathode for achieving a transparent OLED, as shown in Figure 3(e) [49,50]. Such a device is transparent and looks like a window when it is not lit. It can be used as a display or light source upon electrical pumping. Possible applications include double-sided displays or head-up displays. Because of the lack of a highly reflective electrode, it can be understood that the cavity effect is very weak. With suitable material choice and interface engineering, an electrode for simultaneously use as an anode and a cathode can be obtained as a connecting unit for two serial stacking OLEDs, as shown in Figure 3(f)[51]. Such a connecting unit can be made conductive by using metal or metal oxide materials [52,53]. It can be also insulative with a tunneling diode configuration [54]. Hence, there are two or multiple separate recombination zones in such a tandem devices. If a semitransparent electrode is used, one may further consider the coupled cavity effect in such a device, which increases the complexity in device design. In the following, we will introduce the device design of bottom- and top-emission OLEDs.

### 3.1. Bottom-Emission OLEDs

In a bottom emission OLED, because the refractive index of organic layers and ITO (n = 1.6–2.0) are higher than that of glass substrate (n = 1.5) and the thickness of organic layers and ITO are much thinner than that of glass substrate, extraction efficiency can be derived from classical optics as 1/2n_Org_^2^, approximately [55]. Here, n_Org_ represents the refractive index of the organic layer. A simple calculation indicates that extraction efficiency is about 20% with n_Org_ = 1.6. However, when we look closer at the layer structure of the organic layers and ITO, we can see that these layers are as thin as the wavelength of the visible range. Hence, we may regard it as a Fabry-Perot cavity and the emission intensity in the normal direction can be described by the following equation [56]:

(1) $$|E_{\text{out}}^{\text{up}}\left(\lambda \right){|}^{2}=|E_{\text{in}}\left(\lambda \right){|}^{2}\times \frac{T_{2}\times \left[1+R_{1}+2\sqrt{R_{1}}\;\text{cos}(\frac{4\pi x}{\lambda })\right]}{1+R_{1}R_{2}-2\sqrt{R_{1}R_{2}}\text{cos}(\frac{4\pi L}{\lambda })}$$

Here, ${\left|E_{\text{out}}^{\text{up}}(\lambda )\right|}^{2}$ is the output intensity, |*E_in_* (λ)|^2^ is the free-space EL intensity, *x* is the effective distance between recombination zone and the reflective cathode, and *R*_1_ is the reflectivity of the cathode. Also, *R*_2_ and *T*_2_ are the effective reflectivity and transmittivity of the ITO anode side, respectively. *L* is the total optical length (including anode, organic layers, and cathode) of the cavity. When the electrode materials are chosen (*i.e.*, fixing *R*_1_, *T*_1_, *R*_2_, and *T*_2_ values), one can choose an *x* value at the numerator to maximize the output intensity. Then, one can minimize the denominator by changing the *L* value. Note that *x* also affects *L*. Taking a two-layer OLED (consisting only of HTL and ETL sandwiched by ITO and metal cathode) as an example, one should first tune the ETL thickness, and then adjust the HTL and ITO values to obtain the optimized output intensity. Considering the electrical properties, the relative ITO and HTL thickness can be further varied. Note that Equation (1) is wavelength dependent. That means the optimized device structure for one color may be not suitable for other colors, hence for display applications with red, green, and blue primaries, one may need three different device structures to optimize the optical characteristics. Typically, there are some common layers in red, green, and blue OLEDs to reduce the complexity during device fabrication. For white light applications, device design is more difficult, because the emission spectrum typically covers a wide spectral range [57]. Sometimes, tradeoffs among different colors are necessary to obtain the highest efficiency and good color coordinates. Based on the transfer matrix calculation, the optimized extraction efficiency of a bottom-emission OLED can be as high as 26.4% [58]. Although this value is higher than that calculated from classical optics, it reveals that the light extraction from the device is one of the important bottlenecks for OLED efficiency.

### 3.2. Top-emission OLEDs

Compared to bottom-emission OLEDs, extraction efficiency was improved by 20.8% within a 120° viewing cone in a top-emission OLED due to the removal of the glass substrate mode, provided that the organic layers are the same [59]. As shown in Figure 3(b), a top-emission OLED uses the reflective metal as the anode and a transparent (or semitransparent) cathode. However, there are some material and fabrication issues. One of the criteria for the anode material is a high workfunction. Au exhibits a high workfunction, but a Au thin film looks yellowish, because it absorbs the blue light which may be not suitable for full color applicationa. The workfunction of Ti is also high enough, however, conductivity in this case is low and the optical absorption is high, which is not suitable for the anode reflector. A Ag thin film after oxidation with ozone treatment is an alternative choice which provides a high workfunction [60]. For the cathode material, ITO is a straightforward choice, as shown in Figure 3(c)[61]. However, ion bombardment during ITO sputtering may result in the damage to the organic layer underneath. A lower deposition rate is typically required for obtaining better electrical and optical properties [62]. Besides, the workfunction of ITO is high, which is not suitable for electron injection, so typically an EIL for facilitating electron injection, together with protection of the organic layers from ion bombardment is needed [63,64]. Besides, ITO, an alternative is a thin metal film (such as Ag, Sm, and Ca/Mg), as shown in Figure 3(d). Conductivity of metal is about two orders of magnitude higher than that of ITO. This means that the cathode thickness can be reduced by a hundred times when replacing transparent ITO by thin metal film [65]. It can be expected that the reflection from the semitransparent metal cathode is higher, which results in a stronger microcavity effect. By suitable adjustment of the cavity conditions, one may achieve a higher luminance at a certain viewing direction (ex: normal direction) than in a bottom-emission OLED. However, emission luminance and spectrum shift with different viewing direction due to such a strong cavity effect. Typically, a dielectric layer is added on top of the semitransparent one to adjust the transmission, reflection, and affect the cavity length of the cathode for optimizing the optical intensity and selecting the desired wavelength, as shown in Figure 3(e)[66]. Another cavity at the anode side was also proposed for providing an independent control for the optical parameters in the anode side [67].

## 4. OLED with Corrugated Structure

Many efforts have been done for improving the extraction efficiency. Based on the principle of light refraction, reflection and scattering to reduce the total internal reflection (TIR), several approaches were reported for higher extraction efficiency. As shown in Figure 1, non-planar structures can be fabricated on the “device-side” or “air-side” of the substrate to decouple the organic and substrate modes, respectively. Below we will illustrate in detail these two categories for enhancing extracting efficiency.

### 4.1. Light Extraction from Organic Mode

A sub-micron grating structure is used to diffract the light from the waveguiding mode to the external mode. By fabricating an OLED on a 1-D grating structure, one can extract the waveguiding light in the organic and ITO out of the device due to Bragg scattering [68]. Such a corrugated structure can be also fabricated on organic layers with a suitable fabrication process [69]. Not only is extraction efficiency improved, but the extracted light is polarization dependent. To quantitatively illustrate the emission wavelength at different viewing angles (*θ*) to the normal of the substrate, one may use the Bragg scattering equation:

(2) $$k_{0}\;\text{sin}\;\theta =\pm k_{g}\pm m\frac{2\pi }{\Lambda }$$

where *k*_0_ and *k_g_* are the wavevectors in the air and guided (ITO/organic) modes, respectively, *m* is an integer, and Λ is the grating pitch. A 2-D grating (or photonic crystal) can be also used for improving extraction efficiency [70]. The operating principle of this work is also based on sub-micron diffraction. Besides, the holes on the glass substrate have been filled up with SiNx to obtain a smooth surface for OLED fabrication. However, when the distance between the corrugated structure and the OLED is too far, the resulting extraction efficiency is not so obvious. Increases in the extraction efficiency of over 50% and 80% was achieved experimentally and theoretically, respectively, because the periodic modulation converts the guided waves into external leaky waves. Planarization of devices after fabrication of the periodic structure is one of important issues in this configuration for good device performance, which can be accomplished by using polymer materials in a wet process, since the polymer materials will be spin-coated on the surface and hence a flat surface can be achieved. Also, micro- and nano-structures can be obtained through some chemical methods. In 2004, Fichet *et al.* used self-organized structures by spin coating a polymer layer on the top of a 2-D periodical pattern [71]. More than a two-fold increase in efficiency was observed. The pattern consists of hydrophobic and hydrophilic parts, which results in phase separation in certain regiona. The pitch is around 4 μm which is limited by the microcontact printing process. Höfler *et al.* in 2006 showed the possibility of creating planar surfaces with refractive index change, rather than the corrugated structure which increases the difficulties in fabrication [72]. In this study, a five-fold improvement was achieved for the normal direction. By introducing a photorefractive unit into the hole-transporting polymer, refractive index changes can be achieved with Δn = 0.006 at 540 nm.

**Figure 4.**
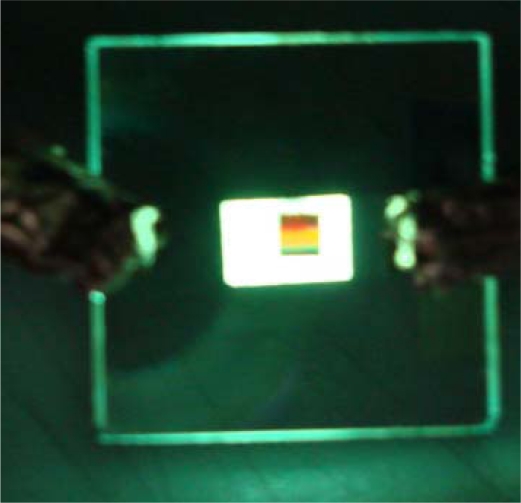
Photo of OLED with 1-D grating.

Surface plasmon resonance (SPR) is another mechanism which can effectively couple the light in organic mode [73]. Although metals are typically opaque, light can transmit though a metal film with nanostructure due to the SPR. By inserting anodic aluminum oxide (AAO) nanoporous films between the ITO transparent electrode and glass substrate, a 50% increase in extraction efficiency was observed [74]. The pore size of AAO is 300 nm, and the cell size is around 400–450 nm. It is interesting that although the metallic hole structure is so thick, it still exhibits a strong extraction efficiency enhancement. In 2007, Chiu *et al.* demonstrated a multi-layer structure upon a 1-D grating to further enhance the light intensity by six times [75,76]. Here, the periodical grating provided an additional in-plane wave vector, and wavevector matching condition of in-plane wavevector (*k_g_*) and surface plasmon wavevector (*k_sp_*) can be obtained. There are two possible surface plasmon modes at the metal/air and metal/organic interfaces, *i.e.*, *k_sp_*_(_*_metal/air_*_)_ and *k_sp_*_(_*_metal/organic_*_)_ respectively, which can be expressed as:

(3) $$k_{g}=k_{\text{organic}}\;\text{sin}\;\theta =\frac{\omega }{c}\sqrt{\frac{{ɛ}_{\text{organic}}{ɛ}_{\text{metal}}}{{ɛ}_{\text{organic}}\;+\;{ɛ}_{\text{metal}}}}\pm m\frac{2\pi }{\Lambda }=k_{sp(\text{metal}/\text{organic})}$$

(4) $$k_{g}=k_{0}\;\text{sin}\;\theta =\frac{\omega }{c}\sqrt{\frac{{ɛ}_{\text{metal}}}{1\;+\;{ɛ}_{\text{metal}}}}\pm m\frac{2\pi }{\Lambda }=k_{sp(\text{metal}/\text{air})}$$

where *k_organic_* is the wavevector in organic material. *ω*, *c*, *ɛ_Alq3_*, *ɛ_m_* are angular frequency, light velocity, relative permittivity of organic emitters and metal, respectively. After designing the layer structures, the PL signals can be as high as 4 and 6 times the original one at certain viewing angles. In this case, tris-(8-hydroxyquinoline) aluminum (Alq3) thin film was employed as the organic emitter which exhibited a broadband emission from 489 to 598 nm centered at 532 nm with the planar structure. Due to the diffraction of the nanostructure, the PL signals were viewing-angle dependent, which changed the emission color from red to blue within 20° viewing angles. Figure 4 shows the photos of EL emission from an OLED with corrugated structure in the upper right region. When such a device was lit, the upper left corner (with corrugated structure) was colorful while the other region (planar structure) emitted a uniform color. The corrugated structure is a 1-D Au grating inserted between the ITO anode and the organic layers. The cathode was a thin metal and this image was taken for the top-side emission.

### 4.2. Light Extraction from Substrate Mode

Gu *et al.* proposed to etch the substrate with truncated cones and fabricate the OLED on the plateau region [77]. Hence, waveguiding light will be reflected by the cone and possibly redirected out of the device. Up to 5.7 and 1.9-fold improvements in extraction efficiency were demonstrated in simulation and experiment, respectively. The increase of the extraction efficiency comes from the light reflection mechanism. However, the active area decreases due to the shaping of the substrate. TIR from high to low refractive index is the root cause of the limitation of the extraction efficiency. That means one may engineer the refractive index value to increase the extraction efficiency. The simplest way to eliminate the glass substrate mode of an OLED is to remove the glass substrate. However, this is impossible because the OLED is too thin and some mechanical support is needed. An alternative way is to insert a very low refractive index material (such as a silica aerogel layer with n = 1.03) between the OLED and the glass substrate [78]. Photons emitted from OLED pass though the aerogel and glass substrate to the air with low, high, and low refractive indexes, respectively. There is no TIR effect and hence one can remove the glass substrate mode with an 80% increase in extraction efficiency. However, the silicon aerogel would be washed out during a wet process, such as ITO etching, which limits its applications. With a non-planar structure on the glass/air interface, one may also reduce the TIR and couple out the glass substrate mode. Monolayers of silica micro-spheres were used as the scattering medium which were put on either sides of the substrate, but not between the OLED or glass substrate [79]. These couple out the light from the substrate mode, rather than the organic mode. Strong scattering light was found from the non-pixel region, which means: (1) it can effectively couple the light out, and (2) image blurring may be a problem when used for display applications. The scattering effect happens between two media with distinct refractive indexes. Although scattering can effectively reduce TIR, it impedes the light outcoupling when the scattering effect is too strong. By controlling the thickness and composition of scattering layer, one may achieve a maximum enhancement of 40% [80,81]. In 2007, Lin *et al.* demonstrated OLED extraction efficiency improvement and light redirection by using a commercial diffuser and brightness enhancement film (BEF) attachment [82]. The diffuser consists of micron range micro-spheres which can effectively scatter the light. Luminance increases for every direction in the diffuser case. On the other hand, BEF is a prism sheet which redirects light with +/–40° to the normal direction. Hence, the light intensity increases most in the normal direction.

**Figure 5.**
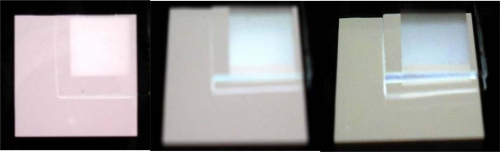
Photos of microlens array film attached on a white OLED observed at different viewing angles: (**a**) 0°, (**b**) 30°, and (**c**) 60°.

**Figure 6.**
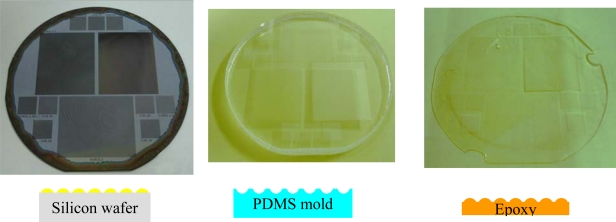
Photos illustrating fabrication of MAF: (**a**) PR on Si wafer, (**b**) PDMS mold, and (**c**) epoxy film.

Similar to the packaging process of a semiconductor LED, a hemispheric dome made of epoxy effectively couples out and redirects light. The attachment of a small OLED on a big hemispheric lens can also effectively couple out the waveguiding mode [83]. However, such a non-planar structure is too bulky. Microlense array film (MAF) was used for the same propose [84]. Figure 5 shows the photos with the MAF attached on a bottom-emission OLED with white-light emission. The MAF was attached on the upper-right corner. One can see a clear enhancement in extraction efficiency in the microlense region of the MAF. Enhancement of extraction efficiency at different viewing angles is 28.3, 54.0, and 49.5% at 0°, 30°, and 60° to the substrate normal. However, due to the light ray redirection, it also resulted in image blurring for display applications. Using a micro-cylinder may result in lower blur [85]. It is also possible to combine the microcavity effect and microlense structure [86]. SiNx/SiOx provides a high reflectivity and hence the cavity effect is strong. In such a microcavity, the color shift at different viewing angle is typically serious, since the effective cavity length varies with different viewing angles. With the attachment of the microlense array, which effectively redirects the light, the spectral shift with different viewing angles is reduced a lot. At the same time, the extraction efficiency improvement is still as high as 80%. There are different methods to fabricate the micro- and nano-structures. In 2004, Wei *et al.* proposed the use of a thermal reflow of the photoresist (PR) to obtain the hemispheric shape, followed by electroforming and UV-forming to transfer the pattern onto the epoxy film, as shown in Figure 6 [87]. At first, PR was patterned on a 4-inch Si-wafer by lithography. After a high temperature treatment of the wafer over glass transition temperature of PR, the dome-shape was formed, as shown in Figure 6(a), where the dark region was occupied by the PR dome. Then, the pattern was transferred reversely to the polydimethylsiloxane (PDMS) mold by a thermal curing process [Figure 6(b)]. Finally, PDMS was then used to duplicate the epoxy film by UV-curing. Figure 6(c) demonstrate the final epoxy film. Also, in 2006, Sun proposed to use imprint lithography to fabricate microlense array attached on the white OLED, which results in a maximum external quantum efficiency of 14.3% at 900 cd/m^2^, power efficiency of 21.6 lm/W at 220 cd/m^2^, and a color rendering index of 87 [88].

When we look more closely to the intensity profile of the OLED, we can observe that although the microlense couples out the light which is originally trapped inside the substrate due to the total internal reflection between the interface of the glass substrate and the air, the corrugated structure of the microlens also impedes the light outcoupling with the photon emission smaller than the critical angle. For coupling out the light smaller than the critical angle, the microlenses are partially removed, which is called “hollow structure” [89]. By removing the microlenses in the emitting layer region, the luminous efficiency can be greatly improved.

In 2007, Chen *et al.* showed experimentally that a pyramidal array light-enhancing layer (pyramidal ALEL) on an OLED panel enhanced the luminance efficiency with a gain factor of 2.03 [90]. Not only was the extraction efficiency discussed, but the Moire effect was also discussed. Since the pyramid has a square base-shape, different alignments (*x* and *y* displacement, and rotational) result in different effects. Not only micro-scale structures, but nano-structures can also be used to effectively increase the light extraction. In 2007, Cheng *et al.* showed that a polydimethylsiloxane (PDMS) film with micro-mesh surface increases the external quantum efficiency [91]. The micromesh was fabricated from a AAO template. Although AAO is in nano-scale range, it bundles and links with one another to form a micro-scale mesh. Extraction efficiency can be increased by 46% with this mesh.

It is also possible to extract organic and substrate modes at the same time. A low index grid (LIG) was inserted between the ITO anode and organic layers to extract the organic modes, while microlense array was attached outside the glass substrate to extract the substrate waveguiding mode [92]. Compared with the planar OLED, 1.32-, 1.68-, and 2.3-fold enhancement in extraction efficiency were observed for the cases of with LIG, with microlenses, and with LIG+microlenses, respectively. Another way to extract the organic mode is to use a high-index substrate. In 2009, Reineke, *et al*. demonstrated a OLED with efficiency comparable to a fluorescent tube in the journal *Nature* [93]. The main objective of this research was to engineer device substrates and output light coupling. In a traditional glass substrate with a low refractive index of 1.5, device efficiency with PIN structure reaches 30 lm/W at 1,000 cd/m^2^. For the same device structure fabricated on a high refractive index glass substrate, device efficiency is improved to 40 lm/W since matched refractive indices of glass substrate and organic layer can extract light trapped within the organic layer to glass substrate. Using a patterned surface of a high refractive index glass substrate or a refractive index matched half-sphere, one can further extract light from the glass mode, thus leading to about 2- and 3-fold efficiency improvement to 90 and 124 lm/W, respectively.

One may also note that although extraction efficiency can be greatly improved by those corrugated structures and this is suitable for lighting applications, it results however in image blur for display applications at the same time. In a real OLED display, a circular polarizer film is attached to the glass substrate for reducing the electrode reflection and hence improving the contrast ratio [94]. However, with the introduction of microstructures, it is not possible to attach such a film. Hence, for display applications, some absorption layer should be added inside an OLED or placed underneath a transparent OLED for reducing the ambient reflection and maintaining the contrast ratio [95–98].

## 5. Conclusions

In summary, we have reviewed the optical design of planar and non-planar OLEDs. An OLED consists of a stacked thin film, with effective optical length comparable to the visible wavelength. Hence, EL from such a device experiences an interference effect and can be viewed as a Fabry-Perot cavity. By engineering the organic layer thicknesses, together with different anode and cathode structures, it is possible to optimize the optical intensity and select desired emission wavelength. However, there is unavoidable waveguiding light in the organic, ITO, and glass substrate. Non-planar structures can effectively extract out those photons to boost the efficiency. By introducing a corrugated structure at the device side, the organic and SPR mode can be effectively extracted. On the other hand, thin films with microstructures attached on the glass substrate can reduce the TIR and extract out the glass substrate mode.
